# Prevalence and severity of laryngeal collapse in dogs undergoing surgery for brachycephalic obstructive airway syndrome: 80 dogs (2018–2022)

**DOI:** 10.3389/fvets.2025.1601466

**Published:** 2025-05-16

**Authors:** Courtney Gallant, Whitney Phipps

**Affiliations:** ^1^Pieper Memorial Veterinary Center, Middletown, CT, United States; ^2^Schwarzman Animal Medical Center, New York, NY, United States

**Keywords:** brachycephalic obstructive airway syndrome, laryngeal collapse, French bulldog, elongated soft palate, laryngeal saccule, brachycephalic

## Abstract

**Objective:**

This study aimed to determine the incidence and severity of laryngeal collapse in brachycephalic breeds presented for surgical management of brachycephalic obstructive airway syndrome (BOAS). The primary hypothesis was that French Bulldogs would have a higher incidence of laryngeal collapse than the other breeds and a higher stage of laryngeal collapse at presentation. The second hypothesis was that higher stages of laryngeal collapse would be associated with increased perioperative complications.

**Methods:**

Clinical records of dogs referred for surgical intervention of BOAS from a single institution were retrospectively reviewed from January 2018 to December 2022. Signalment, physical examination, stage of laryngeal collapse, surgical procedures, the occurrence of postoperative complications, and short-term outcomes were recorded.

**Results:**

French bulldogs were the most common breed presented (53.75%). Fifteen dogs (18.75%) had no evidence of laryngeal collapse, 46 dogs (57.50%) had stage I laryngeal collapse, 13 dogs (16.25%) had stage II laryngeal collapse, and six dogs (7.50%) had stage III laryngeal collapse. There was no association between breed and stage of laryngeal collapse (*p* = 0.68). Postoperative complications were seen in 25/80 (31.25%) dogs. A higher stage of laryngeal collapse was associated with an increased risk of postoperative complications (*p* < 0.0001), including regurgitation, temporary tracheostomy, oxygen supplementation, and aspiration pneumonia. No association was found between additional procedures performed and the incidence of postoperative complications (*p* = 0.31).

**Discussion:**

French bulldogs were the most common breed seen for BOAS surgery during this period. Dogs with a higher stage of laryngeal collapse were at an increased risk of developing postoperative complications. Owners should be counseled that the risks of surgery may be higher with higher stages of laryngeal collapse. Further studies are indicated to evaluate the long-term prognosis.

## 1 Introduction

Brachycephalic obstructive airway syndrome (BOAS) results from the shortened skull and altered pharyngeal anatomy in brachycephalic dogs (1, 2). Common breeds include French bulldogs, English bulldogs, Pugs, and Boston terriers. Primary components include stenotic nares, an elongated and thickened soft palate, redundant pharyngeal folds, and a hypoplastic trachea (2–5). The increased negative thoracic pressure created during inspiration can lead to secondary changes, including varying degrees of laryngeal collapse (6). Stage I laryngeal collapse is defined as eversion of the laryngeal saccules; in stage II, there is loss of rigidity and medial displacement of the cuneiform processes of the arytenoid cartilage, and in stage III, there is collapse of the corniculate processes of the arytenoid cartilages and loss of the dorsal arch of the rima glottidis (7). In 1982, Harvey reported that 53% of a group of brachycephalic dogs had everted laryngeal saccules, and 31% had collapse of the laryngeal cartilage in addition to everted laryngeal saccules (6, 8). Later studies reported everted laryngeal saccules in 58–66% of brachycephalic dogs (2, 5, 9). Brachycephalic obstructive airway syndrome can give rise to a spectrum of clinical respiratory and gastrointestinal symptoms, including inspiratory stertor, stridor, exercise intolerance, regurgitation, vomiting, syncope, and dyspnea (1–5). This increases the risk of severe respiratory distress requiring emergent medical intervention. The presence of laryngeal collapse has been previously found to be a negative prognostic factor in the surgical treatment of BOAS (10).

The popularity of brachycephalic dogs has been steadily increasing; the AKC registrations for French bulldogs have increased by over 1,000% since 2012 (11). With this increase in popularity, dogs experiencing clinical signs of BOAS are becoming increasingly prevalent, potentially impacting the prevalence of dogs experiencing laryngeal collapse. While many studies have focused on components BOAS, surgical treatment, and prognosis, to the authors’ knowledge, no recent studies have explicitly evaluated the prevalence and severity of laryngeal collapse in dogs using Leonard’s grading system. The objectives of this study are to report the prevalence and severity of laryngeal collapse and evaluate breed distribution in a population of brachycephalic dogs presenting for surgical intervention for BOAS. The primary hypothesis was that French Bulldogs would have a higher incidence of laryngeal collapse than the other breeds and a higher stage of laryngeal collapse at presentation. The second hypothesis was that higher stages of laryngeal collapse would be associated with an increased incidence of perioperative complications.

## 2 Materials and methods

### 2.1 Inclusion/exclusion criteria

Electronic medical records of client-owned brachycephalic dogs presented for brachycephalic obstructive airway surgery from January 2018 to December 2022 were reviewed. Dogs were excluded if they had incomplete medical records or did not undergo a sedated laryngeal examination with a boarded or residency-trained surgeon. Data retrieved from the medical records included age, breed, sex, weight, reason for presentation and evaluation, clinical findings during initial physical examination, presence and grade of laryngeal collapse, surgical procedures performed, duration of hospitalization, occurrence of intraoperative and postsurgical complications until discharge, and short-term outcome. Routine laboratory, thoracic radiographs, and abdominal ultrasound results were reviewed when available. Complications were reviewed within the hospitalization period and the short-term period between discharge and recheck examination up to 20 days post-operative. Postoperative complications were classified on a 0–4 scale based on a modification of the Accordion Severity Classification of Postoperative Complications (see Table 1) (12).

**Table 1:** Definitions of postoperative complications based on a modification of the Accordion Severity Classification of Postoperative Complications.

Level	Definition
0	No complications
1	Mild complication: post-operative regurgitation; <6 h postoperative oxygen supplementation; aspiration pneumonia requiring oral antibiotics, but not extending hospital stay
2	Moderate complication: reintubation not requiring ventilation or tracheostomy; >6 h postoperative oxygen supplementation; aspiration pneumonia requiring injectable antibiotics
3	Severe complication: temporary tracheostomy; mechanical ventilation; aspiration pneumonia or other cause of prolonged hospitalization (>48 h)
4	Post-operative death

### 2.2 Procedures

Grading of laryngeal collapse was performed by a residency-trained or board-certified surgeon via sedated laryngeal examination immediately prior to induction of anesthesia using the system described by Leonard: stage I: laryngeal saccule eversion; stage II: laryngeal saccule eversion and cuneiform process of arytenoid cartilage collapse; stage III: corniculate process of arytenoid cartilage collapse (7). Dogs were recorded as having stage I collapse for cases where everted laryngeal saccules were recorded in the medical record, but a specific laryngeal collapse stage was not recorded. Cases recorded as a range or intermediate stage were classified as the higher of the two stages (i.e., stage I-II was recorded as stage II).

Sedation and anesthetic protocol for laryngeal examination were at the discretion of the attending clinician. In general, this included pre-medication with an alpha-2 agonist and induction with intravenous propofol to effect. Doxapram was not routinely utilized for the laryngeal examination of brachycephalic patients at this institution.

Surgical procedures were recorded as a single procedure or a combination of alarplasty, staphylectomy, and laryngeal sacculectomy. The decision regarding which surgical technique and combination of procedures to perform was clinician-dependent and was based on surgeon preference and upper airway evaluation. Anesthetic protocol and postoperative treatment were at the discretion of the attending surgeon.

### 2.3 Data analysis

Data collected from the medical records were tabulated, and percentages for each item were calculated. Descriptive statistics are presented as mean ± SD for normally distributed variables or as median and range for non-normally distributed variables. One-way frequency tables (ANOVA for normally distributed data and Kruskal Wallis for non-normally distributed data) and summary statistics were developed using a commercially available software program (NCSS 2023, Kaysville, UT). The χ^2^ test was used for associations between breed, procedures performed, laryngeal collapse stage, and complications. Values of *p* ≤ 0.05 were considered significant. Weighted κ agreement statistics were used to test for associations between grade severity of laryngeal collapse and complications to take ordered scores into account.

## 3 Results

Eighty-three dogs presented for brachycephalic obstructive airway surgery during the study period. Eighty dogs met the inclusion criteria for the study. There were 27 (33.75%) intact males, 29 (36.25%) neutered males, seven (8.75%) intact females, and 17 (21.25%) spayed females. Breeds represented included 43 (53.75%) French bulldogs, 20 (25.00%) English bulldogs, seven (8.75%) Pugs, four (5.00%) Boston terriers, and six (7.50%) other breeds, including one Shih Tzu, and five mixed breed dogs. The median age was 36 months, ranging from 5 to 146 months. Median body weight was 14.7 kg, ranging from 7.1 kg to 38.4 kg.

Sixty-nine (89.25%) dogs had a history of increased respiratory noise, 34 (42.50%) dogs had gastrointestinal signs (regurgitation or vomiting), 4 (5.0%) dogs had a prior history of aspiration pneumonia, and 2 (2.50%) dogs had no owner reported BOAS clinical signs.

Fifteen dogs (18.75%) had no evidence of laryngeal collapse, 46 dogs (57.50%) had stage I laryngeal collapse, 13 dogs (16.25%) had stage II laryngeal collapse, and six dogs (7.50%) had stage III laryngeal collapse (Table 2). No dogs were documented to have laryngeal paralysis. There was no association between breed and stage of laryngeal collapse (*p* = 0.68). Laryngeal collapse was absent in the two dogs with no reported BOAS clinical signs.

**Table 2:** Distribution of dog breeds in the study population and stage of laryngeal collapse.

	Stage 0 (%)	Stage I (%)	Stage II (%)	Stage III (%)	Total
French bulldog	7 (16.28)	25 (65.12)	8 (18.60)	3 (6.98)	43
English bulldog	2 (10.00)	13 (65.00)	3 (15.00)	2 (10.00)	20
Pug	1 (14.28)	4 (57.14)	1 (14.28)	1 (14.28)	7
Boston terrier	2 (50.00)	2 (50.00)	0	0	4
Other	3 (50.00)	2 (33.33)	1 (16.67)	0	6
Total	15 (18.75)	46 (57.5)	13 (16.25)	6 (7.50)	80

The procedures performed are summarized in Table 3. Two (2.50%) dogs had no airway procedure performed, four (5.0%) dogs had alarplasty, two (2.50%) dogs had staphylectomy, 10 (12.50%) dogs had alarplasty and staphylectomy, 14 (17.50%) dogs had staphylectomy and laryngeal sacculectomy, 48 (60.00%) dogs had alarplasty, staphylectomy, and laryngeal sacculectomy. Of the 15 dogs with no laryngeal collapse, one (6.67%) dog had no airway procedure performed, four (26.67%) dogs had alarplasty only, two (13.33%) dogs had staphylectomy only, eight (53.33%) dogs had alarplasty and staphylectomy. Of the 46 dogs with stage I laryngeal collapse, 10 (21.74%) dogs had staphylectomy and laryngeal sacculectomy, 36 (78.26%) dogs had alarplasty, staphylectomy, and laryngeal sacculectomy. Of the 13 dogs with stage II laryngeal collapse, one (7.69%) dog had no procedures, two (15.38%) dogs had alarplasty and staphylectomy, one (7.69%) dog had staphylectomy and laryngeal sacculectomy, nine (69.23%) dogs had alarplasty, staphylectomy and laryngeal sacculectomy. Of the six dogs with stage III laryngeal collapse, three (50%) dogs had staphylectomy and laryngeal sacculectomy, three (50.00%) dogs had alarplasty, staphylectomy and laryngeal sacculectomy. Sixty-five dogs presented with everted laryngeal saccules. Out of these dogs, 62 (95.38%) underwent laryngeal sacculectomy. There was an association between the procedure performed and the stage of laryngeal collapse (*p* < 0.0001). Dogs with more severe laryngeal collapse received more surgical procedures. There was no association between BOAS surgical procedures performed and the incidence of postoperative complications (*p* = 0.58). Thirty-five (43.75%) dogs had one or more non-BOAS surgeries performed concurrently. This included 29 (36.25%) castrations or ovariohysterectomies, five (6.25%) mass excisions, two (2.50%) orthopedic procedures, and one (1.25%) each of the following: entropion correction, umbilical hernia repair, tibial plateau leveling osteotomy, total ear canal ablation and bulla osteotomy, and liver biopsy. Forty-five (56.25%) dogs underwent only BOAS-related procedures. No association was found between additional procedures performed and the incidence of postoperative complications (*p* = 0.31).

**Table 3:** Summary of airway procedures performed for each of the dog breeds in the study population.

	None (%)	Alarplasty (%)	Staphylectomy (%)	Alarplasty + Staphylectomy (%)	Staphylectomy + Sacculectomy (%)	Alarplasty + Staphylectomy + Sacculectomy (%)	Total
French bulldog	1 (2.32)	1 (2.32)	1 (2.32)	5 (11.63)	7 (16.28)	28 (65.12)	43
English bulldog	0	0	1 (5.00)	2 (10.00)	4 (20.00)	13 (65.50)	20
Pug	1 (14.28)	1 (14.28)	0	0	2 (28.57)	3 (42.86)	7
Boston terrier	0	1 (25.00)	0	1 (25.00)	1 (25.00)	1 (25.00)	4
Other	0	1 (16.67)	0	2 (33.33)	0	3 (50.00)	6
Total	2 (2.50)	4 (5.00)	2 (2.50)	10 (12.50)	14 (17.50)	48 (60.00)	80

The median number of nights spent in hospital postoperatively was one night, ranging from 0 to 11. The overall complication rate was 25/80 (31.25%) dogs. There was no association between sex, castration status, age, or weight, and the development of postoperative complications (*p* = 0.36, 0.99, 0.63, 0.56, respectively). French bulldogs and English bulldogs were the only breeds to experience postoperative complications. Of the 20 English bulldogs in the study, 14 (70.0%) had no complications, five (25.00%) had level 1 complications, and one (5.00%) had level 4 complications. Among the 43 French Bulldogs, 24 (55.81%) had no complications, 12 (27.91%) had level 1 complications, four (9.30%) had level 2 complications, and three (6.98%) had level 3 complications. The English bulldog with level 4 complications had stage III laryngeal collapse, and underwent cardiopulmonary arrest 8 days postoperative alarplasty, staphylectomy, laryngeal sacculectomy, and temporary tracheostomy, and 4 days postoperative permanent tracheostomy after continued respiratory distress and the development of tricavitary effusion. There was no association between these two breeds and the development of postoperative complications (*p* = 0.38).

There was a positive correlation between dogs with more severe laryngeal collapse and the incidence of postoperative complications (*p* = 0.0009). Among the dogs with no laryngeal collapse, 12 (80.00%) dogs had no complications, and three (20.00%) had level 1 complications. Among the dogs with stage I laryngeal collapse, 33 (71.74%), 10 (21.74%), two (4.35%), and one (2.17%) dogs had levels 0, 1, 2 and 3 complications, respectively. Among the dogs with stage II laryngeal collapse, eight (61.54%), three (23.08%), and two (15.38%) dogs had levels 0, 1, and 2 complications, respectively. Among the dogs with stage III laryngeal collapse, two (33.33%), one (16.67%), two (33.33%), and one (16.67%) dogs had levels 0, 1, 3, and 4 complications, respectively. Four dogs (5.00%) required a temporary tracheostomy postoperatively, three of which had stage III laryngeal collapse and one with stage I laryngeal collapse. The risk of having a level 3 complication was 15 times greater in dogs with stage III laryngeal collapse compared to stage I.

## 4 Discussion

In this study, 81.25% of dogs were affected by some degree of laryngeal collapse, with 23.75% having stage II or greater, although the breed was not associated with the presence of laryngeal collapse or stage of laryngeal collapse at presentation. Therefore, the hypothesis that French bulldogs would have a higher incidence of laryngeal collapse than the other breeds and a higher stage of laryngeal collapse at presentation is rejected. The presence of stage II and III laryngeal collapse in this study population is higher than in previous studies that ranged from 2 to 23% (3, 5, 9, 13), but is similar to a recent study by Fracka where 31% of dogs had stage II or greater laryngeal collapse (14).

The most common breed in this study population was the French bulldog (53.75%); this is higher than in previous reports (4–42%) (2, 3, 5, 10, 13). Historically, Pugs have been among the most prevalent breeds presenting with BOAS (21–42%), with up to 40% of these having stage III laryngeal collapse (15). In this study, Pugs made up a small portion of dogs presenting for airway surgery (5.0%), and only one was found to have stage III laryngeal collapse. This may represent differences in regional breed distribution or reflect the overall increase in French bulldog popularity in recent years, along with the selection for progressively shorter muzzles.

In this study population, a higher stage of laryngeal collapse was associated with a higher incidence of complications. Therefore, the second hypothesis was accepted. Reported postoperative complications of BOAS surgery include regurgitation, coughing, dyspnea, cyanosis, airway edema and swelling, respiratory tract obstruction, and aspiration pneumonia (7). We chose to classify postoperative complications based on a modification of the Accordion Severity Grading System to organize reported complications by invasiveness of therapy needed (12). The requirement of oxygen supplementation for less than 6 h after anesthetic recovery was chosen as the cutoff for mild complications. This time point was chosen as it will differentiate between dogs able to be transitioned to room air by the end of the clinical day and those requiring oxygen supplementation overnight.

We found a significant association between dogs with a higher stage of laryngeal collapse and an increased incidence of postoperative complications. This contrasts previous reports that found that postoperative complications and surgical outcome were not associated with the stage of laryngeal collapse (13, 15) but is in agreement with the study by Liu et al., which showed that the stage of laryngeal collapse was a negative prognostic indicator (10). A possible explanation is that dogs with stage II or III laryngeal collapse will have a more narrow airway postoperatively due to the collapse of the corniculate and/or cuneiform cartilages that are not addressed. Additionally, these patients may experience uncoordinated or paradoxical motion of their larynges, further contributing to dynamic airway obstruction post-operatively. Postoperative swelling in these cases is more likely to occlude the airway, resulting in an upper airway obstruction, the need for oxygen supplementation, and ongoing gastrointestinal clinical signs.

Hughes et al. reported that dogs undergoing laryngeal sacculectomy in addition to nares resection and staphylectomy were more likely to experience postoperative complications (regurgitation, coughing, dyspnea, death) compared to those undergoing nares resection and staphylectomy alone (16). This finding brought into question the necessity of laryngeal sacculectomy for everted laryngeal saccules. However, this study did not consider the effect of laryngeal collapse on the incidence of postoperative complications. Within our study population, there was an association between a higher stage of laryngeal collapse and an increased rate of postoperative complications. The overall complication rate in our study of 31% was similar to the complication rate of 33% reported by Hughes. However, we found no association between the surgical procedures performed and the development of postoperative complications. Only three of the 65 dogs in our population that presented with everted laryngeal saccules did not undergo laryngeal sacculectomy. This makes the effect of laryngeal sacculectomy unable to be directly evaluated in this population. However, a higher complication rate in this study would be expected if an increased rate of complications was associated with laryngeal sacculectomy alone.

This study has several limitations, which are related to its retrospective nature. There was no standardization of the medical records, which could have resulted in incomplete records and possibly some minor complications being underreported. Multiple board-certified surgeons (one achieving diplomate status during the study period) had primary responsibility for each case over the study period. Treatments were based on clinician discretion, which may have resulted in differences in surgical procedures and postoperative management. Similarly, case management may have been transitioned to other surgeons or critical care specialists during hospitalization, resulting in differences in management and record keeping. Despite the standardized grading scheme, multiple observers likely also caused minor variations in reported laryngeal collapse stages. Another limitation of this study is the small number of each breed with stage III laryngeal collapse and the low rate of severe postoperative complications, which may have led to a type II error. *Post hoc* power analysis revealed that a larger sample size is needed to detect a significant difference between breeds and the development of postoperative complications. To detect a significant difference in the development of level 3 complications between French Bulldogs and other breeds with 80% power, then 68 French Bulldogs and 68 other breeds would be required. A prospective study with standardized surgical procedures in a larger population of dogs is needed to assess the effects of breed and stage of laryngeal collapse on outcome following BOAS surgery.

In conclusion, this study suggests that some degree of laryngeal collapse is present in most dogs presenting for BOAS surgery and may be of a higher frequency and stage than previous reports suggest. Owners should be educated that dogs with a higher stage of laryngeal collapse are at a higher risk of developing postoperative complications. Further studies with larger case numbers are needed to evaluate breed associations and long-term outcomes of dogs with laryngeal collapse.

## Data Availability

The raw data supporting the conclusions of this article will be made available by the authors, without undue reservation.
